# Peripheral immune tolerance alleviates the intracranial lipopolysaccharide injection-induced neuroinflammation and protects the dopaminergic neurons from neuroinflammation-related neurotoxicity

**DOI:** 10.1186/s12974-017-0994-3

**Published:** 2017-11-16

**Authors:** Yang Liu, Xin Xie, Li-Ping Xia, Hong Lv, Fan Lou, Yan Ren, Zhi-Yi He, Xiao-Guang Luo

**Affiliations:** Department of Neurology, The First Affiliated Hospital of China Medical University, 155 North Nanjing Street, Heping District, Shenyang, 110001 People’s Republic of China

**Keywords:** Microglia, Peripheral blood monocytes, Neuroinflammation, Neuroprotection, Neurodegeneration disease

## Abstract

**Background:**

Neuroinflammation plays a critical role in the onset and development of neurodegeneration disorders such as Parkinson’s disease. The immune activities of the central nervous system are profoundly affected by peripheral immune activities. Immune tolerance refers to the unresponsiveness of the immune system to continuous or repeated stimulation to avoid excessive inflammation and unnecessary by-stander injury in the face of continuous antigen threat. It has been proved that the immune tolerance could suppress the development of various peripheral inflammation-related diseases. However, the role of immune tolerance in neuroinflammation and neurodegenerative diseases was not clear.

**Methods:**

Rats were injected with repeated low-dose lipopolysaccharide (LPS, 0.3 mg/kg) intraperitoneally for 4 days to induce peripheral immune tolerance. Neuroinflammation was produced using intracranial LPS (15 μg) injection. Inflammation cytokines were measured using enzyme-linked immunosorbent assay (ELISA) and quantitative real-time polymerase chain reaction (qRT-PCR). Microglial activation were measured using immunostaining of Iba-1 and ED-1. Dopaminergic neuronal damage was evaluated using immunochemistry staining and stereological counting of TH-positive neurons. Behavioral impairment was evaluated using amphetamine-induced rotational behavioral assessment.

**Results:**

Compared with the non-immune tolerated animals, pre-treatment of peripheral immune tolerance significantly decreased the production of inflammatory cytokines, suppressed the microglial activation, and increased the number of dopaminergic neuronal survival in the substantia nigra.

**Conclusions:**

Our results indicated that peripheral immune tolerance attenuated neuroinflammation and inhibited neuroinflammation-induced dopaminergic neuronal death.

**Electronic supplementary material:**

The online version of this article (10.1186/s12974-017-0994-3) contains supplementary material, which is available to authorized users.

## Background

Neuroinflammation is one of the protuberant pathological features of neurodegenerative diseases, including Parkinson’s disease (PD) [1]. PD is characterized by a dramatic loss of dopaminergic (DA) neurons in the substantia nigra (SN). Characteristic features of neuroinflammation include the activation of glial cells and a series of inflammatory mediators including pro-inflammatory cytokines, chemokines, reactive oxygen species, and nitric oxide [2]. These elements have been proved to involve in the development of neuronal degeneration [3].

Despite the protection of the blood-brain barrier (BBB), the central nervous system (CNS) could be deeply affected by the peripheral immune. It is well established that peripheral inflammation can give rise to neuroinflammation and is linked closely to the etiology and progress of neurodegenerative diseases [4, 5]. One famous study showed that a single intraperitoneal (i.p.) injection of lipopolysaccharide (LPS) resulted in an elevated production of pro-inflammatory cytokines and the delayed loss of dopaminergic neurons in the SN [6]. On the other hand, long-term use of non-steroidal anti-inflammatory drugs could decrease the incidence of PD [7, 8]. These results suggest that the neuroinflammation can be strongly influenced by the peripheral inflammation.

Microglia, as the vital component of the brain immune system, plays a crucial role in maintaining brain microenvironment homeostasis [9]. Once activated, it can transform from resting state into activated state by changing its morphology, proliferating and initiating the inflammatory response by producing pro-inflammatory cytokines and neurotoxic mediators such as nitric oxide, prostaglandin E2, and reactive oxygen species [10] and by cross-talking with other glial cells, BBB cells, and neurons [11, 12]. Studies have revealed that microglial over-activation caused by inflammation may disrupt the microenvironment in the brain and pose a threat to the dopaminergic neuronal survival.

Monocytes, as the promoter and organizer of the peripheral innate immune, play multiple roles in the immune system. Recently, a growing body of research is concentrated on the role of monocytes play in disorders of the CNS [13]. One of our recent studies showed that depletion of peripheral blood monocytes (PBM) attenuated not only peripheral inflammation but also neuroinflammation induced by peripheral LPS injection. Our results demonstrated that monocytes are the peripheral decisive elements in the process of neuroinflammation induced by peripheral inflammation [14]. Moreover, monocyte and microglia are considered to share the same origin of the myeloid lineage [15, 16]; mounting evidence reveals that monocytes could infiltrate into the brain and develop into microglia [17] or construct a special group of macrophages [18]. These findings imply that the immune response of peripheral blood monocytes is of profound significance to the progression and prognosis of the neuroinflammation.

Immune tolerance refers to the refractoriness of the immune system when suffering continuous antigen threat. It is a protective mechanism of the body with suppressed peripheral immune activities in order to avoid excessive inflammatory response and unnecessary by-stander injury [19]. Endotoxic tolerance (ET) induced by a low dose of endotoxin (lipopolysaccharide, LPS) is deemed as the most essential component of immune tolerance [20]. Remarkably, LPS peripheral preconditioning has been proved to alleviate the process of several neurological disorders, such as middle cerebral artery occlusion syndrome (MCAO) [21], brain trauma [22], and brain injury [23]. These results demonstrate the important role immune tolerance plays in neuroprotection.

Immune tolerance can inhibit peripheral immune activity and reduce subsequent inflammation; however, little is known about its role in neurodegenerative diseases. Here, we proposed that the pre-treatment to induce peripheral immune tolerance can inhibit intracranial LPS injection-induced neuroinflammation and neurodegeneration. These results may provide a novel therapeutic strategy for treating neuroinflammation-related neurodegenerative diseases.

## Methods

### Animals

Experiments were conducted on 112 male Sprague-Dawley rats (8 weeks old, 220–240 g, Animal Center of China Medical University), according to the National Institute of Health Guidelines (National Institutes of Health, Bethesda, USA) and approved by the ethics committee of China Medical University. Animals had unrestricted access to food and water and were housed in groups (4 rats per cage) in a temperature-controlled environment with an ambient temperature of 22 °C (± 1 °C) and a 12-h light-dark cycle.

### Experimental procedures and grouping

**Fig. 1:**
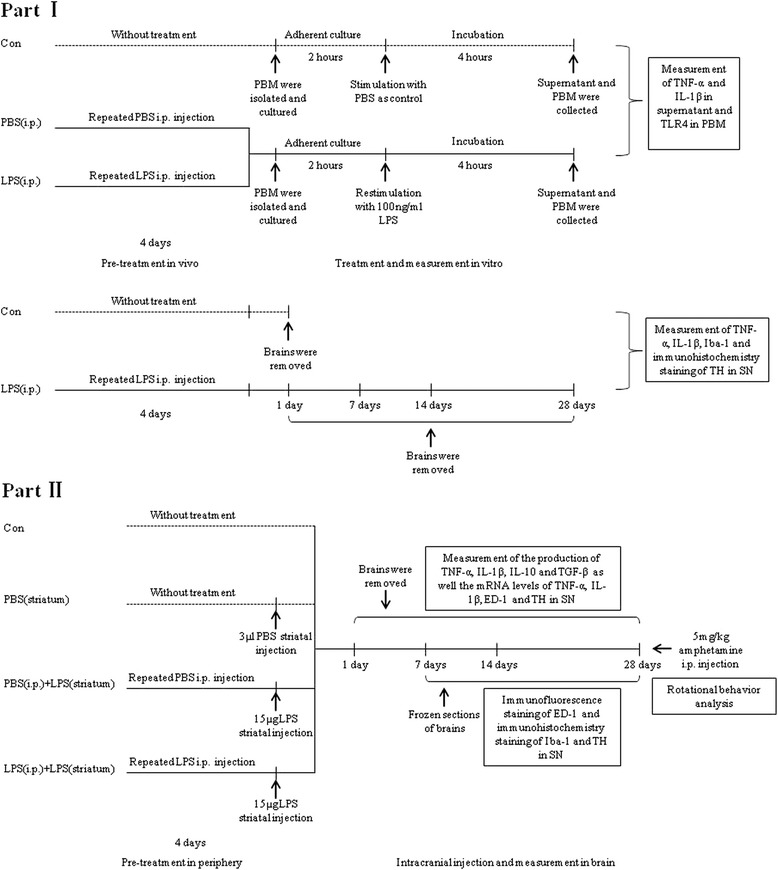
Study procedure and grouping. In the first part of the experiment, we confirmed that repeated low-dose LPS i.p. injection could induce immune tolerance of PBM without inflammation or dopaminergic neuronal loss in the brain. To induct endotoxic tolerance, rats were pre-treated with repeated 0.3 mg/kg LPS intraperitoneal injection for 4 days. Then, PBM were isolated, cultured, and restimulated by a hige-dose LPS. Moreover, we verified that peripheral repeated low-dose LPS intraperitoneal administration was failing to cause inflammation or dopaminergic neuronal loss in the brain parenchyma of rats. According to the different preconditioning, three groups were involved: the control group (*n* = 15), the PBS (i.p.) group (*n* = 6), and the LPS (i.p.) group (*n* = 42). In the second part of the experiment, we investigated the neuroprotective role of peripheral immune tolerance in intracranial LPS injection-induced neuroinflammation-related neurodegeneration. Neuroinflammation-induced PD rat model was conducted by 15 μg LPS intracranial injection into the right striatum. Inflammatory cytokines, microglial activation, loss of dopaminergic neurons, as well the behavior impairment were detected at indicated time points. Four groups were involved, including a control group without treatment, a PBS(striatum) group with striatal PBS injection as the sham group, a PBS (i.p.) + LPS (striatum) group with peripheral PBS pre-treatment prior to striatal 15 μg LPS injection, and a LPS (i.p.) + LPS (striatum) group with peripheral LPS pre-treatment prior to striatal 15 μg LPS injection

Before treatment, animals were numbered and acclimated for 1 week. Afterwards, rats were randomly divided into the following groups according to a random number table. Experimental procedures and grouping was shown as Fig. 1. In part **I**, three groups were involved: (1) a control group without treatment (control group, *n* = 15), (2) a group treated with repeated intraperitoneal (i.p.) injections of phosphate buffered saline (PBS) for 4 days [PBS (i.p.) group, *n* = 6], and (3) a group treated with repeated i.p. injections of 0.3 mg/kg LPS for 4 days [LPS (i.p.) group, *n* = 42]. In part **II**, four groups were involved: (1) a control group without treatment (control group, *n* = 16), (2) a sham group treated with intracranial PBS injection into the right striatum [PBS (striatum) group, *n* = 16], (3) a group treated with repeated i.p. injections of PBS for 4 days plus an intracranial LPS injection into the right striatum [PBS (i.p.) + LPS (striatum) group, *n* = 16], and (4) a group treated with repeated i.p. injections of LPS for 4 days plus an intracranial LPS injection into the right striatum [LPS (i.p.) + LPS (striatum) group, *n* = 16].

### Induction and verification of peripheral immune tolerance

LPS (strain O111; B4, Sigma, St Louis, USA) was dissolved in PBS (pH 7.4) at a concentration of 0.1 mg/ml. To generate peripheral immune tolerance, rats were given pre-treatment with repeated i.p. injections of LPS (0.3 mg/kg body weight/single/day) for 4 days. (A variety of concentrations have been explored in the preliminary experiment; afterwards, the dose of LPS at 0.3 mg/kg was determined ultimately. Additional file 1: Figure S1.) After the peripheral pre-treatment, rats were sacrificed for peripheral blood, and then, peripheral blood monocytes (PBM) were cultured with combined density gradient centrifugation and attachment culture method. 1 × 10^6^ cells/ml was regarded as the appropriate cell density for incubation at 37 °C under 5% CO_2_. After the adherent cell growth, medium was changed, and 100 ng/ml LPS was given to PBM in vitro for 4 h in order to mimic subsequent inflammation. To test the immunological competence of PBM, the levels of pro-inflammatory cytokines (TNF-α and IL-1β) in the supernatant were measured by enzyme-linked immunosorbent assay (ELISA), while also the expression of toll-like receptor 4 (TLR4) in monocytes was measured by Western blot.

### Stereotaxic surgery

For stereotaxic surgery, rats were anesthetized with an i.p. injection of chloral hydrate (400 mg/kg). Afterwards, they were placed in a stereotaxic apparatus. The skull was exposed, and a dental drill was used to make a small hole to allow for the striatal injection. LPS (15 μg in 3 ul, dissolved in PBS) was injected unilaterally at the following coordinates: AP, − 0.4 mm; *L*, + 3.0 mm; *V*, − 5.5 mm, which corresponded to the largest segment of striatum in the coronal plane [24]. (In our preliminary experiments, we explored LPS-induced PD rats model with various stereotaxic injection sites and various doses of LPS; ultimately, unilateral striatal injection of 15 μg LPS was adopted as the PD model in our present study. Additional file 2: Figure S2.) The sham group was injected with vehicle (PBS, 3 μl). The injection was controlled at a rate of 1 ul/min, and the Hamilton syringe needle was left in place for 5 min and then slowly withdrawn to prevent reflux of the solution.

### Measurement of cytokines

#### Supernatant of cultured PBM

At 4 h from 100 ng LPS treatment, supernatant was collected and the amount of TNF-α and IL-1β was measured with ELISA techniques. ELISA was performed according to manufacturer’s instructions (R&D Company, Minneapolis, USA). The detection limits of TNF-α and IL-1β were 800 and 50 pg/ml.

#### SN of rat brain

Rats were sacrificed 1, 7, 14, and 28 days after the final treatment. The right SN were dissected out on ice. The amount of pro-inflammation cytokines (TNF-α, IL-1β) and anti-inflammation cytokines (TGF-β, IL-10) from SN was measured with ELISA techniques. Tissues were homogenized in 1000 ul of ice-cold PBS (0.01M, pH 7.4) and centrifuged at 12,000 g at 4 °C for 15 min. Centrifugal supernatant was collected, and ELISA was then performed according to manufacturer’s instructions (R&D Company, Minneapolis, USA). The detection limit of TNF-α, IL-1β, IL-10, and TGF-β were 300, 80, 50, and 200 pg/ml, respectively.

### Western blot analysis

#### Cultured PBM

At 4 h from 100 ng LPS treatment, cultured PBM was scraped and collected with 150 ul of ice-cold lysis buffer (1 ml RIPA Lysis Buffer, 10 μl PMSF, Biyotime, Shanghai, China) for 30 min splitting and then centrifuged (12,000 g at 4 °C for 15 min). Centrifugal supernatant was kept at − 80 °C until used for analysis.

#### SN of rat brain

Rats were sacrificed 1, 7, 14, and 28 days after the final treatment, and the right SN were dissected out on ice. Tissues were homogenized in 1000 ul of ice-cold lysis buffer (1 ml RIPA Lysis Buffer, 10 μl PMSF, Biyotime, Shanghai, China) and lysed at 4 °C for 30 min. The resultant lysates were centrifuged (12,000 g at 4 °C for 30 min) to remove particulate materials. Centrifugal supernatant was kept at − 80 °C until used for analysis.

Afterwards, protein concentration was determined using the BCA kit (Biyotime, Shanghai, China) and enzyme analyzer. Equal amounts of proteins (15 μg for cell lysates; 30 μg for tissue lysates) were separated by 10% SDS-PAGE gels and transferred to polyvinylidene difluoride (PVDF) membranes (Bio-Rad, California, USA) using an electrophoretic transfer system (Bio-Rad, California, USA), which were blocked in 5% fat-free milk (room temperature, 2 h). The membranes were incubated in primary antibodies (mouse antibody against TLR4, Abcam, Danvers, USA, 1:400; goat antibody against Iba-1, Wako, Tokyo, USA, 1:800; mouse antibody against GAPDH, Abcam, Danvers, USA, 1:1000) overnight at 4 °C. Then, the membranes were rinsed with tris-buffered saline containing 0.1% tween 20 (TBST) 3 times for 10 min per time before incubation in secondary antibody (horseradish peroxidase-labeled anti-mouse IgG, Abcam, Danvers, USA, 1:2000; horseradish peroxidase-labeled anti-goat IgG, Abcam, Danvers, USA, 1:2000) for 2 h at room temperature. This was followed by treatment with the ECL chemiluminescent reagents (Bio-Rad, California, USA) and exposed using the ECL chemiluminescence detector (Bio-Rad, California, USA). A density measurement for each band was performed with the ImageJ software. The relative levels of the TLR4 and Iba-1 were normalized against the levels of GAPDH and expressed graphically.

### Quantitative real-time PCR

Rats were sacrificed, and the right SN was immediately isolated. The total RNA from the right SN was extracted using Trizol (Takara, Dalian, China) according to manufacturer’s instructions and was then quantified photometrically. Reverse transcription was performed with the PrimeScript™ RT Reagent Kit (Takara, Dalian, China). Quantitative real-time PCR (qRT-PCR) was conducted using the SYBR Premix Ex Taq™ (Takara, Dalian, China) and a Light Cycler 480 II Real-Time PCR system (Roche Diagnostics, Basel, Switzerland). The relative expression among the groups was calculated using the 2^−ΔΔCt^ method. The primer sequences were listed as the following: rat GAPDH forward, GCAGCCCAGAACATCATCC reverse, GTCATCATACTTGGCAGGTT; rat TNF-α forward, ACTGAACTTCGGGGTGATTG reverse, GTGGGTGAGGAGCACGTAGT; rat IL-1β forward, AGCTGCACTGCAGGCTTCGAGATG reverse, GAACTGTGCAGACTCAAACTCCAC; rat ED-1 forward, TGAGGTTCCCTGTGTGTCTG reverse, GGTTGTAGGTGTCTCCGTGAA; rat TH forward, CTACTGTCCGCCCGTGATTT reverse, CACAGGCTGGTAGGTTTGATC.

### Tissue preparation

At 1, 7, 14, and 28 days post the striatal injection, the rats were deeply anesthetized and perfused through the aorta with 0.9% saline, followed by 4% paraformaldehyde dissolved in PBS (0.01M, pH 7.4). Brains were dissected and fixed in 4% buffered paraformaldehyde and cryoprotected in 30% sucrose solution at 4 °C for 2 days. Coronal serial sections (20 um) throughout the SN were cut using a freezing microtome (CM1900, Leica, Germany) maintained at − 20 °C. Afterwards, the sections were stored in 65% glycerinum at − 20 °C for histological analysis.

### Immunohistochemistry and immunofluorescence

Brain sections were rinsed with PBS and pre-treated with 0.3% H_2_O_2_ for 15 min to inhibit endogenous peroxidase activity, and then rinsed in PBS 3 times for 5 min per time. Then, the sections were blocked with 0.5% bovine serum albumin (BSA) for 30 min at room temperature. Sections were incubated overnight at 4 °C with the following primary antibodies: mouse antibody against ED1 (CD68, Abcam, Danvers, USA, 1:400), goat antibody against tyrosine hydroxylase (TH, Abcam, Danvers, USA, 1:800), goat antibody against Iba-1 (Wako, Tokyo, USA, 1:800). The sections were washed with PBS post to the incubation. For fluorescence staining, sections were incubated with Alexa Fluor 594-conjugated secondary antibodies (Molecular Probes, California, USA, 1:1000) for 2 h at room temperature. Besides, Hoechst (Biyotime, Shanghai, China) was used to label the cell nucleus. Immunostaining sections were evaluated using a fluorescence microscope (Olympus BX51, Tokyo, Japan). For histochemistry staining, sections were incubated with a biotinylated rabbit-anti-goat secondary antibody for 40 min after the incubation of primary antibodies, followed by incubation with avidin-conjugated horseradish peroxidase for 40 min. DAB was used to develop the color. The sections were dehydrated using an alcohol gradient, cleared in xylene, and cover slipped with neutral gum for microscopic analysis.

### Stereological counting of dopaminergic neurons

To evaluate the loss of dopaminergic neurons, the unbiased stereological counting of the TH-positive neurons was made using the optical fractionator method performed on an Olympus Computer Assisted Stereological Toolbox system, version 2.1.4 (Olympus, Ballerup, Denmark). This sampling technique was previously described in detail with some modifications [25, 26], which is unaffected by the volume of reference or the size of the counted elements [27]. The coronal serial sections (20 μm) used for counting covered the entire SN, from the rostral tip of the pars compacta (AP, − 4.30 mm) back to the caudal end of the pars reticulate (AP, − 6.72 mm). This generally yielded about 120 sections in each rat. For the estimation of the total numbers of TH-positive neurons in the SN, every sixth section was counted; moreover, a counting frame of 40 × 40 μm and a grid size of 150 × 150 μm was used. Guard volume (4 μm from the top, 4–6 μm from the bottom of the section) was excluded from both surfaces to avoid the problem of lost cap, and only the profiles that came into focus within the counting volume (with a depth of 10 μm) were counted. The estimate of the total numbers of TH-positive neurons was calculated according to the optical fractionator formula [27].

### Behavioral assessment

At 28 days, test for amphetamine-induced rotational behavior was carried out. For the test, rats were placed in a hemispherical bowl immediately after receiving 5 mg/kg amphetamine (dissolved in normal saline, Sigma, St Louis, USA) i.p. injections. The number of ipsilateral 360° turns was measured every minute for 180 min per rat.

### Statistical analysis

Data are presented as the mean ± standard error of the mean (SEM). Tests of variance homogeneity, normality, and distribution were performed to ensure that the assumptions required for standard parametric analysis of variance were satisfied. When the homogeneity of variance was equal, one-way analysis of variance (ANOVA) followed by Least Significant Digit (LSD) test was used. When the assumption of homogeneity of variances is not met, Welch’s test was applied and Dunnett T3 test was used for comparison of pairwise significance. All data were analyzed using SPSS 19.0, and *P* values less than 0.05 were considered statistically significant.

## Results

### Repeated low-dose LPS intraperitoneal injection-induced endotoxic tolerance of PBM

**Fig. 2:**
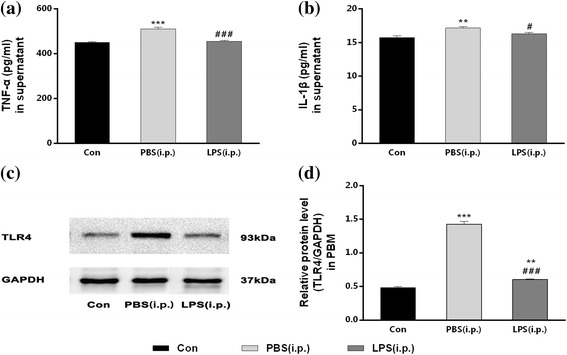
Repeated low-dose LPS intraperitoneal injection-induced endotoxic tolerance of PBM. PBM from three groups with or without LPS pre-treatment in vivo were isolated and cultured. Then, TNF-α and IL-1β secreted by PBM and TLR4 expressed in PBM were quantified post to a single high-dose LPS (100 ng/ml) administration for 4 h in vitro. The productions of TNF-α and IL-1β were decreased and the expression of TLR4 in PBM was downregulated after LPS restimulation by repeated 0.3 mg/kg LPS intraperitoneal injection in LPS (i.p.) group. **a**, **b** The levels of TNF-α (**a**) and IL-1β (**b**) in the supernatant were detected by ELISA. **c**, **d** The expression of TLR4 in PBM was quantified by measuring band intensities using ImageJ software. The values were normalized to GAPDH. **P < 0.01, ***P < 0.001 vs. control group. #*P* < 0.05, ###*P* < 0.001 vs. PBS (i.p.) group. The data are presented as the mean ± SEM (*n* = 6)

To test whether endotoxic tolerance could be induced by repeated low-dose LPS intraperitoneal injection, TNF-α and IL-1β secreted by PBM and TLR4 expressed in PBM were quantified post to a single-dose LPS (100 ng/ml) administration. In the PBS (i.p.) group, which received repeated intraperitoneal injection of PBS prior to a single high dose of LPS administration, the TNF-α and IL-1β amounts in the supernatant were both increased; moreover, the expression of TLR4 was also upregulated, compared with the control group (without LPS restimulation), as shown in Fig. 2. Compared to the PBS (i.p.) group, a significant decrease in the levels of TNF-α (10.91%, *P* < 0.01) and IL-1β (5.04%, *P* < 0.05) were observed in the LPS (i.p.) group, which was caused by repeated 0.3 mg/kg LPS intraperitoneal injection. Besides, the TLR4 expression was upregulated by 25.71% (*P* < 0.01) in the LPS (i.p.) group than in the control group, but it was still downregulated by 57.57% (*P* < 0.01), compared with the PBS (i.p.) group. These results demonstrated that endotoxic tolerance of PBM could be induced by repeated low-dose LPS intraperitoneal injection in rats.

### Repeated low-dose LPS intraperitoneal injection was not sufficient to cause inflammation in the SN

**Fig. 3:**
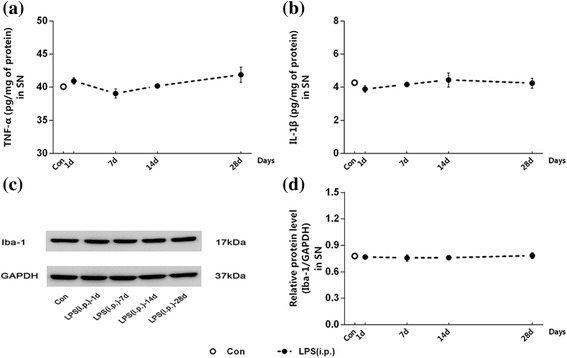
Repeated low-dose LPS intraperitoneal injection preconditioning did not cause inflammation in the SN. We investigated the levels of pro-inflammatory cytokines and the expression of Iba-1 in the right SN of rats at 1, 7, 14, and 28 days post to the peripheral LPS injection. There was no significant difference between the control group and any other time points of the LPS (i.p.) group in the amounts of TNF-α and IL-1β nor in the expression of Iba-1. **a**, **b** The levels of TNF-α (**a**) and IL-1β (**b**) in the right SN were detected by ELISA. **c**, **d** The expression of Iba-1 in the right SN was quantified by measuring band intensities using ImageJ software. The values were normalized to GAPDH. The data are presented as the mean ± SEM (*n* = 6)

To verify that the pre-treatment with repeated low-dose LPS intraperitoneal injection was not sufficient to cause inflammation in the SN, we investigated the levels of pre-inflammatory cytokines (TNF-α and IL-1β) and the expression of Iba-1 in the right SN of rats from the LPS (i.p.) group at 1, 7, 14, and 28 days post to the last peripheral LPS injection. As showed in Fig. 3, there was no significant difference between the control group and any other time points of the LPS (i.p.) group, neither in the amounts of TNF-α (Fig. 3a), IL-1β (Fig. 3b) nor in the expression of Iba-1 (Fig. 3c, d). Time-dependent effect did not exist either. The results indicated that repeated low-dose LPS intraperitoneal injection was not sufficient to cause inflammation in the SN of rats.

### Repeated low-dose LPS intraperitoneal injection was not sufficient to cause dopaminergic neuronal loss in the SN

**Fig. 4:**
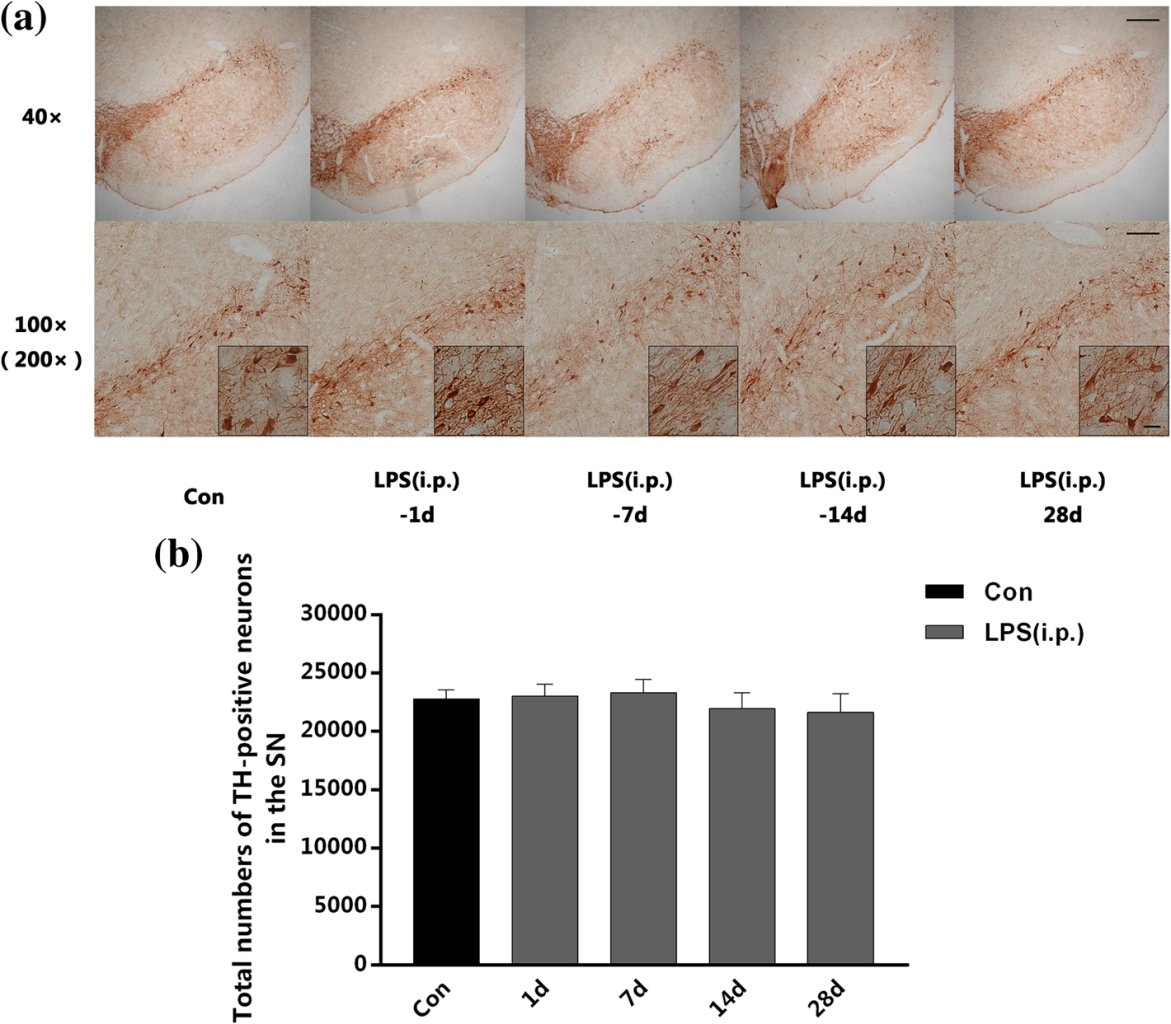
. Repeated low-dose LPS intraperitoneal injection preconditioning did not cause dopaminergic neuronal loss in the SN. We carried out immunohistochemical staining of TH and stereological counting of TH-positive neurons in the non-intracranial injected SN of rats at 1, 7, 14, and 28 days after the peripheral LPS injection. There was no significant differences between the control group and any other time points of the LPS (i.p.) group in the total numbers of TH-positive cells. **a** Immunohistochemistry staining on frozen sections of TH in the control group and any other time points of the LPS (i.p.) group. **b** Total numbers of TH-positive neurons in the right SN collected by stereological counting (*n* = 3, each group). The data are presented as the mean ± SEM. Scale bars, 250 μm (40×), 100 μm (100×), and 25 μm (200×)

To confirm that the pre-treatment with repeated low-dose LPS intraperitoneal injection was not sufficient to cause dopaminergic neuronal loss in the SN, we carried out immunohistochemical staining of TH and stereological counting of TH-positive neurons in the non-intracranial injected SN of rats from the LPS (i.p.) group at 1, 7, 14, and 28 days post to the last peripheral LPS injection. As showed in Fig. 4, dopaminergic neuronal morphology (Fig. 4a) and the total numbers of TH-positive cells (Fig. 4b) expressed no significant difference between the control group and any other time points of the LPS (i.p.) group. The results indicated that repeated low-dose LPS intraperitoneal injection was not sufficient to cause dopaminergic neuronal loss in the SN of rats.

### Peripheral immune tolerance preconditioning decreased intracranial LPS injection-induced pro-inflammatory cytokines in the SN

**Fig. 5:**
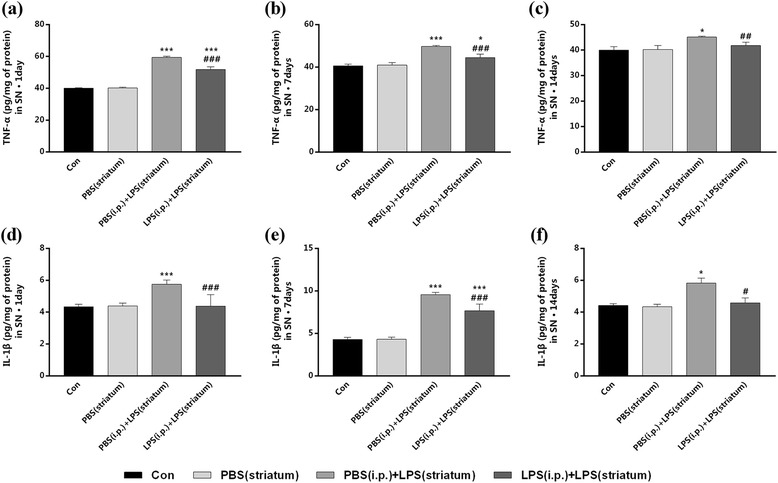
Peripheral immune tolerance preconditioning decreased intracranial LPS injection-induced production of TNF-α and IL-1β in the SN. Rats from four groups with or without peripheral LPS pre-treatment were sacrificed, and right SN was dissected out at time point of 1, 7, 14, and 28 days after striatal LPS injection for ELISA analysis. The results showed that the production of TNF-α and IL-1β were decreased by peripheral LPS preconditioning at 1, 7, and 14 days after the intracranial LPS exposure. **a–f** The expression of TNF-α (**a–c**) and IL-1β (**d–f**) in the right SN were detected by ELISA at 1, 7, and 14 days. The difference was imperceptible at 28 days, so the data was not shown.*P < 0.05, ***P < 0.001 vs. control group. ##*P* < 0.01, ###*P* < 0.001 vs. PBS (i.p.) + LPS (striatum) group. The data are presented as the mean ± SEM (*n* = 7)

To test whether peripheral immune tolerance preconditioning was able to prevent intracranial LPS injection-induced neuroinflammation, we first investigated the expressions of pro-inflammatory cytokines (TNF-α and IL-1β) in the right SN by ELISA. Striatal LPS administration caused a time-dependent increase of TNF-α and IL-1β expressions in the SN both in the groups with or without peripheral LPS preconditioning (Fig. 5). Compared with the PBS (i.p.) + LPS (striatum) group, the endotoxic tolerant pre-treatment significantly decreased the expression of TNF-α by 12.86, 10.45, and 7.35% (Fig. 5a–c), as well as decreased the expression of IL-1β by 23.84, 19.68, and 21.42% at 1, 7, and 14 days individually post to the striatal LPS injection (Fig. 5d–f).

**Fig. 6:**
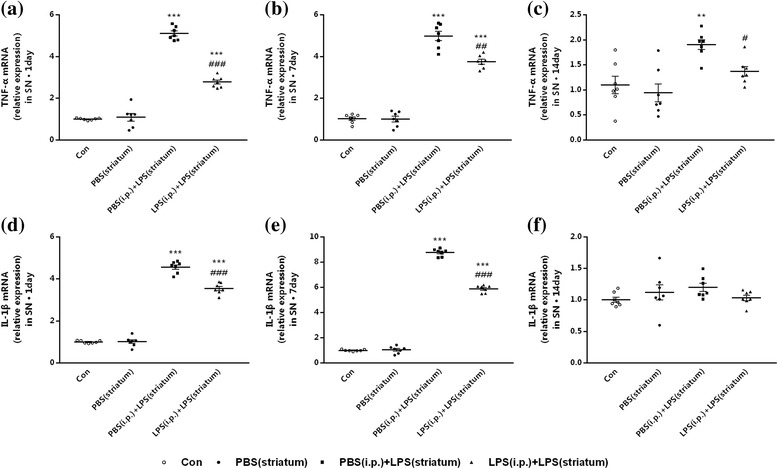
Peripheral immune tolerance preconditioning downregulated intracranial LPS injection-induced expression profiles of TNF-α and IL-1β in the SN. Rats from four groups with or without peripheral LPS pre-treatment were sacrificed, and right SN was dissected out at time point of 1, 7, 14, and 28 days after striatal LPS injection for qRT-PCR analysis. The results showed that peripheral immune tolerance preconditioning downregulated the expression profiles of TNF-α at 1, 7, and 14 days, as well as the expression profiles of IL-1β only at 1 and 7 days after the intracranial LPS exposure. **a–c** The mRNA levels of TNF-α in the right SN were detected by qRT-PCR. **d–f** The mRNA levels of and IL-1β in the right SN were detected by qRT-PCR. *P < 0.05, **P < 0.01, ***P < 0.001 vs. control group. #*P* < 0.05, ##*P* < 0.01, ###*P* < 0.001 vs. PBS (i.p.) + LPS (striatum) group. The data are presented as the mean ± SEM (*n* = 7)

Then, we examined the expression profiles of TNF-α and IL-1β in the right SN by qRT-PCR. Significant decreases in the mRNA levels of TNF-α and IL-1β were observed in the LPS (i.p.) + LPS (striatum) group compared to the PBS (i.p.) + LPS (striatum) group at indicated time points (Fig. 6). The LPS (i.p.) + LPS (striatum) group showed significant decreased TNF-α mRNA levels at 1, 7, and 14 days (Fig. 6a–c), as well as decreased IL-1β mRNA levels at 1 and 7 days (Fig. 6d–f) individually post to the striatal LPS injection, compared with the PBS (i.p.) + LPS (striatum) group. Striatal vehicle injection [PBS (i.p.) group] made no differences in the amounts of pro-inflammatory cytokines compared with the control group.

The results showed that peripheral immune tolerance preconditioning decreased intracranial LPS injection-induced pro-inflammatory cytokines in the SN.

### Peripheral immune tolerance preconditioning did not alter intracranial LPS injection-induced anti-inflammatory cytokines in the SN

**Fig. 7:**
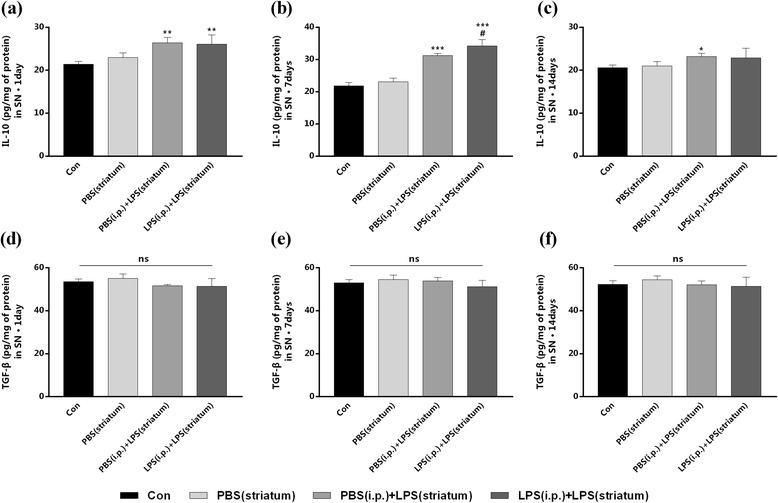
Peripheral immune tolerance preconditioning did not alter intracranial LPS injection-induced production of IL-10 and TGF-β in the SN. Rats from four groups with or without peripheral LPS pre-treatment were sacrificed, and right SN was dissected out at time point of 1, 7, 14, and 28 days after striatal LPS injection for ELISA analysis. The results indicated that the production of IL-10 was increased at 7 days after the intracranial LPS exposure, while there were no differences in our observation of IL-10 at any other time points as well as TGF-β at any time points between the PBS (i.p.) + LPS (striatum) group and the LPS (i.p.) + LPS (striatum) group. **a–f** The expression of IL-10 (**a–c**) and TGF-β (**d–f**) in the right SN were detected by ELISA. *P < 0.05, **P < 0.01, ***P < 0.001 vs. control group. #*P* < 0.05 vs. PBS (i.p.) + LPS (striatum) group. The data are presented as the mean ± SEM (*n* = 7)

To test whether peripheral immune tolerance preconditioning was able to prevent intracranial LPS injection-induced increase of anti-inflammatory factors, we measured the expressions of anti-inflammatory cytokines (IL-10 and TGF-β) in the right SN by ELISA. The IL-10 level increased and lasted at least 14 days after the striatal LPS injection in both groups with or without peripheral LPS preconditioning (Fig. 7a–c). However, the endotoxic tolerance pre-treatment only made significant increase of IL-10 by 9.54% at 7 days post to the striatal LPS exposure (Fig. 7b).

Furthermore, there were no significant difference in the expression of TGF-β between the LPS (i.p.) + LPS (striatum) group and the PBS (i.p.) + LPS (striatum) group at any time points of our observation (Fig. 7d–f). Striatal vehicle injection [PBS (i.p.) group] made no differences in the amounts of anti-inflammatory cytokines compared with the control group.

These results suggested that peripheral immune tolerance preconditioning did not alter intracranial LPS injection-induced anti-inflammatory cytokines in the SN.

### Peripheral immune tolerance preconditioning inhibited intracranial LPS injection-induced microglial activation in the SN

**Fig. 8:**
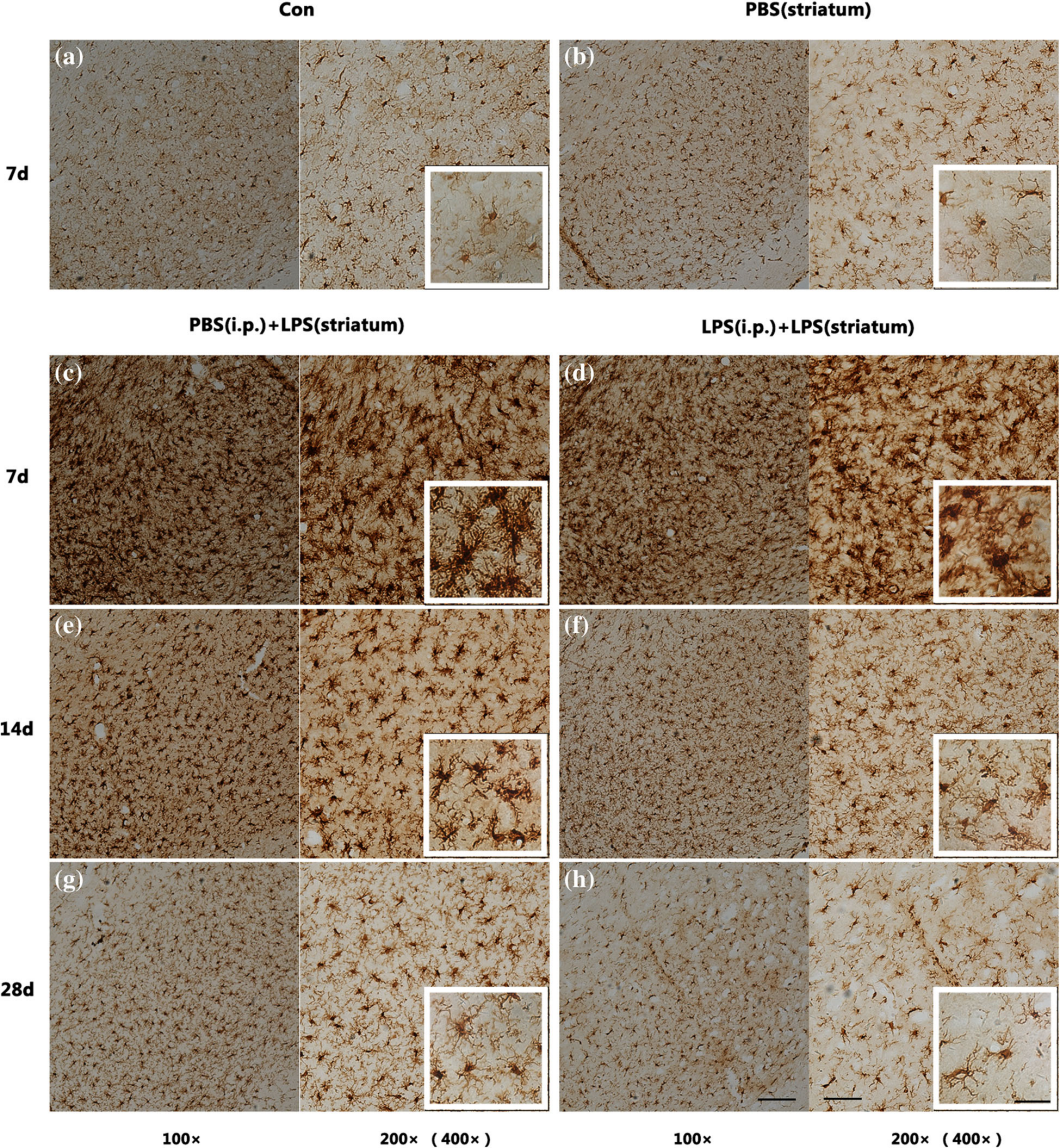
Comparison of microglial morphology in the SN among different pre-treated groups. The results displayed that intracranial LPS injection caused microglial activation respectively characterized by “amoeboid form” at 7 days (**c**, **d**) or “typical activated form” at 14 days (**e**, **f**) post to the LPS exposure. However, endotoxic tolerant preconditioning resulted in fewer microglial counting and lighter immunohistochemical staining (**d**, **f**, and **h**). Since the control group and the PBS (striatum) group were not statistically significant at each time point, we selected 7 days as a representative (**a**, **b**). Scale bars, 100 μm (100×), 50 μm (200×), and 25 μm (400×)

Firstly, we observed morphological changes of microglia using immumohistochemical staining of Iba-1. As showed in Fig. 8, microglia in the control group were “resting form” characterized by small somas, thin processes, as well as a small cellular counting (Fig. 8a). At 7 days post to the LPS exposure, amoeboid microglia had been observed in right SN (Fig. 8c, d). While microglia were “typical activated form” characterized by enlarged somas and thickened processes at 14 days post to the LPS administration (Fig. 8e, f). However, endotoxic tolerant preconditioning still resulted in fewer microglial counting and lighter immunohistochemical staining (Fig. 8d–h). Microglia in the LPS (i.p.) + LPS (striatum) group had almost returned to resting state at 28 days (Fig. 8h), while some of microglia in the PBS (i.p.) + LPS (striatum) group had still showed deeper immunohistochemical staining (Fig. 8g), compared with the control group. Striatal vehicle injection [PBS (i.p.) group] did not alter microglial morphology (Fig. 8b), compared with the control group.

Nextly, we measured the expression of ED-1 in the SN by immunofluorescence assay.

**Fig. 9:**
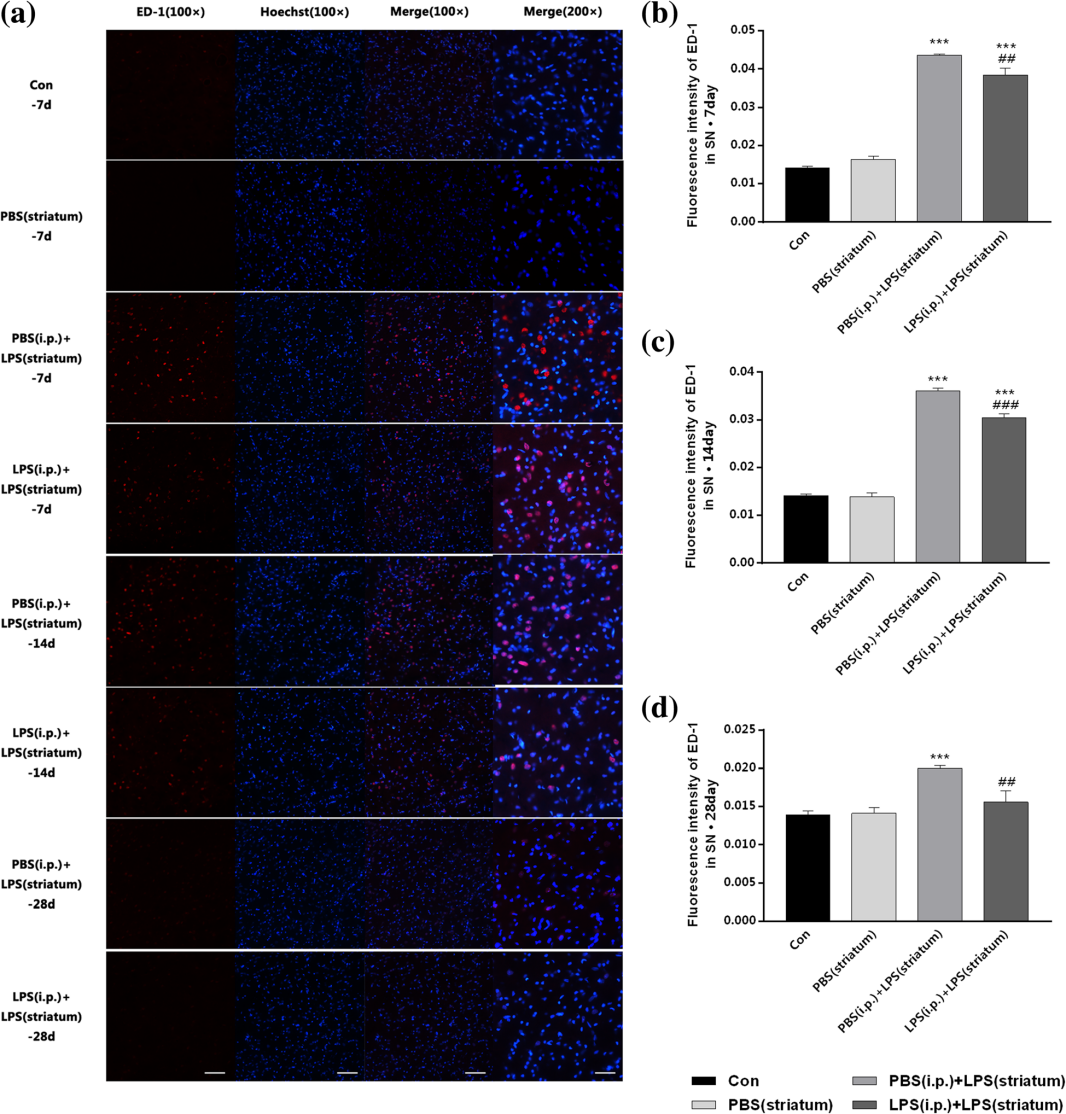
Intracranial LPS injection-induced microglial activation was inhibited by peripheral immune tolerance preconditioning in the SN. Rats from four groups with or without peripheral LPS pre-treatment were sacrificed, and then, the brains were removed at 7, 14, and 28 days after striatal injection. The results displayed that peripheral immune tolerance pre-treatment relieved intracranial LPS injection-induced microglial activation with decreased fluorescence intensity of ED-1. **a** Immunofluorescence staining on frozen sections of ED-1 (*red*) and Hoechst (*blue*) in the four groups of our observation. Since the control group and the PBS (striatum) group were not statistically significant at each time point, we selected 7 days as a representative. **b–d** The bar chart to display the fluorescence intensity of ED-1 (*n* = 3, each group). ***P < 0.001 vs. control group. ##*P* < 0.01, ###*P* < 0.001 vs. PBS (i.p.) + LPS (striatum) group. The data are presented as the mean ± SEM. Scale bars, 100 μm (100×) and 50 μm (200×)

As shown in Fig. 9, compared with control group, both two groups received intracranial LPS injection showed a time-dependent enhancement of fluorescent intensity post to the striatal LPS exposure. Compared with the PBS (i.p.) + LPS (striatum) group, the pre-treatment with endotoxic tolerance decreased the fluorescent intensity by 11.65% (Fig. 9b). A decreased fluorescent intensity of ED-1 was presented in both two groups at 14 days; furthermore, the peripheral LPS pre-treatment reduced the fluorescent intensity by 15.41% (Fig. 9c). Moreover, the fluorescence intensity of ED-1 in the LPS (i.p.) + LPS (striatum) group mainly returned to the normal state at 28 days, while it was still 1.41 times higher in the PBS (i.p.) + LPS (striatum) group than in the control group.

**Fig. 10:**
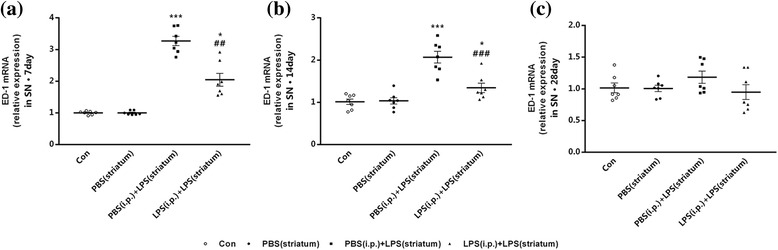
Intracranial LPS injection-induced microglial activation was inhibited by peripheral immune tolerance preconditioning in the SN. Rats from four groups with or without peripheral LPS pre-treatment were sacrificed, and then, the brains were removed at 7, 14, and 28 days after striatal injection. The results displayed that peripheral immune tolerance pre-treatment relieved intracranial LPS injection-induced microglial activation with downregulated mRNA levels of ED-1. **a–c** The mRNA levels of ED-1 were quantified by qRT-PCR (*n* = 7, each group). *P < 0.05, ***P < 0.001 vs. control group. ##*P* < 0.01, ###*P* < 0.001 vs. PBS (i.p.) + LPS (striatum) group. The data are presented as the mean ± SEM

Then, the expression profiles of ED-1 in the right SN were detected by qRT-PCR. The ED-1 mRNA level showed a time-dependent increase in both two groups received intracranial LPS injection, with a maximum at 7 days and a minimum at 28 days post to the striatal LPS administration (Fig. 10a–c). In addition, pre-treatment with endotoxic tolerance decreased the ED-1 mRNA level at 7 days (*P* < 0.01) (Fig. 10a) and 14 days (*P* < 0.01) (Fig. 10b), but did not significantly alter the ED-1 expression profiles at 28 days (Fig. 10c). Striatal vehicle injection [PBS (i.p.) group] made no differences in the ED-1 expression compared with the control group.

The results showed that endotoxin tolerance preconditioning inhibited intracranial LPS injection-induced microglial activation in the SN.

### Peripheral immune tolerance pre-treated PD rats showed decreased intracranial LPS injection-induced dopaminergic neuronal loss in the SN

Unilateral striatal injection of 15 μg LPS made a progressive and significant loss of the TH-positive neurons: 29.78% at 14 days, 54.59% at 28 days after the striatal 15 μg LPS exposure; however, no differences have been detected at 1 or 7 days, compared with the group receiving striatal 3 μl PBS exposure (Additional file 2: Figure S2).

**Fig. 11:**
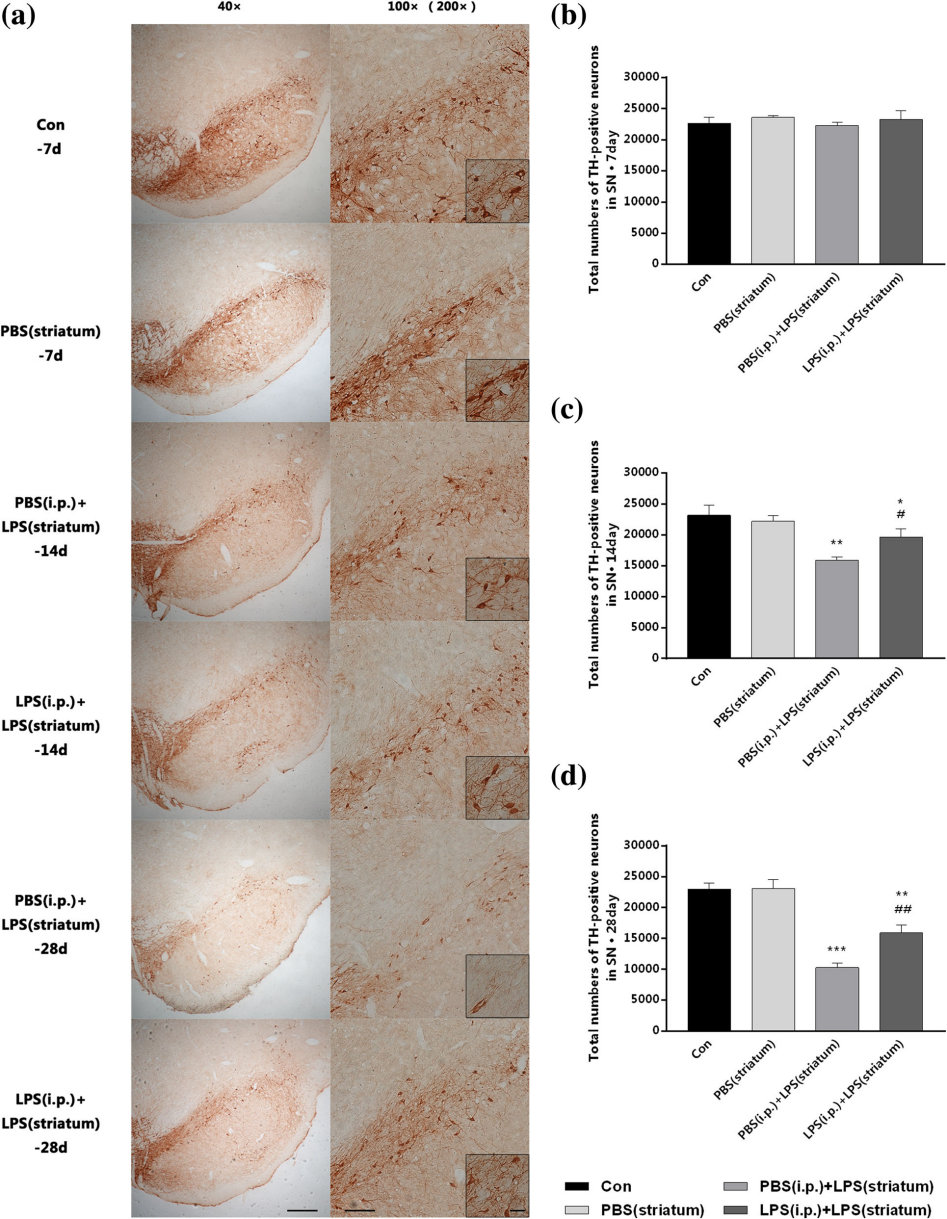
Peripheral immune tolerance pre-treated PD rats were protected from intracranial LPS injection-induced neurodegeneration with increased dopaminergic neuronal survival in the SN. Rats from four groups with or without peripheral LPS pre-treatment were sacrificed, and then, brains were removed at 7, 14, and 28 days after striatal injection. The results implied that peripheral immune tolerance pre-treatment protected the PD rats from neuroinflammation-related neurodegeneration with increased dopaminergic neuronal survival. **a** Immunohistochemistry staining on frozen sections of TH in the four groups of our observation. Since the control group and the PBS (striatum) group were not statistically significant at each time point, we selected 7 days as a representative. Moreover, the difference had not been distinguished as early as 7 days, so the data of the PBS (i.p.) + LPS (striatum) group and the LPS (i.p.) + LPS (striatum) group at 7 days was not shown. **b–d** Total numbers of TH-positive neurons in the injected side of SN (right SN) collected by stereological counting (*n* = 3, each group). *P < 0.05, **P < 0.01, ***P < 0.001 vs. control group. #*P* < 0.05, ##*P* < 0.01 vs. PBS (i.p.) + LPS (striatum) group. The data are presented as the mean ± SEM. Scale bars, 250 μm (40×), 100 μm (100×), and 25 μm (200×)

To probe whether endotoxic tolerance pre-treated PD rats could decrease neuroinflammation-induced dopaminergic neuronal loss, we carried out immunohistochemical staining of TH and stereological counting of TH-positive neurons in the SN. As showed in Fig. 11, the striatal LPS injection decreased the total numbers of TH-positive neurons over time in both two groups; however, this reduction was more rapid and severe in group without endotoxic tolerance preconditioning. Peripheral LPS preconditioning made a 23.67% increase in the total numbers of TH-positive neurons (Fig. 11c). Moreover, the neuroprotection became more prominent at 28 days, with an increase of 54.64% (Fig. 11d).

**Fig. 12:**
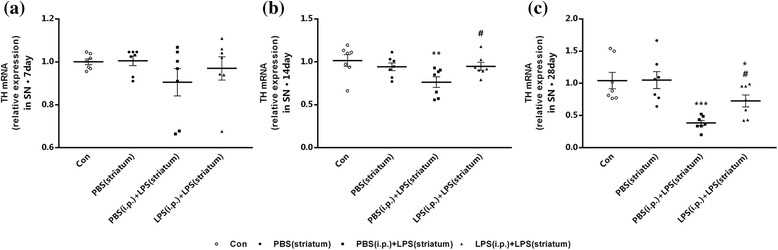
Peripheral immune tolerance pre-treated PD rats were protected from intracranial LPS injection-induced neurodegeneration with increased dopaminergic neuronal survival in the SN. Rats from four groups with or without peripheral LPS pre-treatment were sacrificed, and then, the brains were removed at 7, 14, and 28 days after striatal injection. The results implied that peripheral immune tolerance pre-treatment protected the PD rats from neuroinflammation-related neurodegeneration with upregulated mRNA levels of TH. **a–c** The mRNA levels of TH were quantified by qRT-PCR (*n* = 7, each group). **P < 0.01, ***P < 0.001 vs. control group. #*P* < 0.05 vs. PBS (i.p.) + LPS (striatum) group. The data are presented as the mean ± SEM

The expression profiles of TH in the right SN were then detected by qRT-PCR. The TH mRNA level of PBS (i.p.) + LPS (striatum) group showed a more obvious decrease over time, with LPS (i.p.) + LPS (striatum) group as a control (Fig. 12a–c). At 14 days, the TH mRNA level in two groups received striatal LPS administration were 0.763 and 0.948 times of control group (Fig. 12b), it revealed that endotoxic tolerance pre-treatment upregulated the TH mRNA level (*P* < 0.01); moreover, the difference became more obvious at 28 days (Fig. 12c), while the difference was not shown at 7 days (Fig. 12a, b). Striatal vehicle injection [PBS (i.p.) group] made no differences in the TH expression compared with the control group.

These results underlined that endotoxic tolerance pre-treatment could decrease intracranial LPS injection-induced dopaminergic neuronal loss.

### Peripheral immune tolerance pre-treated PD rats showed improved intracranial LPS injection-induced behavioral impairment

**Fig. 13:**
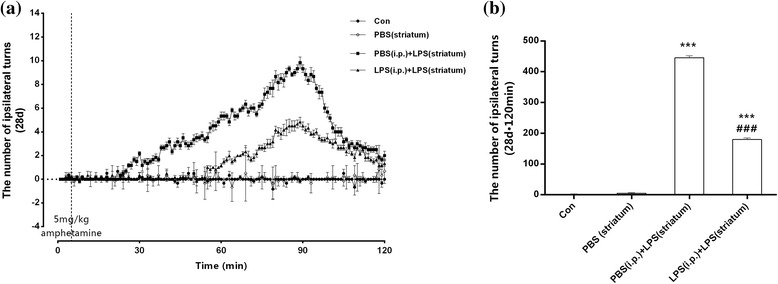
Peripheral immune tolerance pre-treated PD rats were protected from intracranial LPS injection-induced neurodegeneration with improved behavioral impairment. Rats from the control group, the PBS (striatum) group, the PBS (i.p.) + LPS (striatum) group, and the LPS (i.p.) + LPS (striatum) group were administrated a single dose of amphetamine (5 mg/kg) on day 28 post to intracranial injection, and the numbers of ipsilateral turns were recorded with 120 min. The results showed alleviated behavioral impairment caused by peripheral LPS pre-treatment. **a** The numbers of ipsilateral turns per minutes for 120 min after the amphetamine injection. **b** The total numbers of ipsilateral turns within 120 min after the amphetamine injection. ***P < 0.001 vs. control group. ###*P* < 0.001 vs. PBS (i.p.) + LPS (striatum) group. The data are presented as the mean ± SEM (*n* = 6)

Test for amphetamine-induced rotational behavior was performed at 28 days post to the striatal LPS injection to assess the behavioral impairment. As shown in Fig. 13, striatal LPS injection caused a marked ipsilateral rotational behavior toward the lesion side in the PBS (i.p.) + LPS (striatum) group. A noticeable decrease (59.65%, *P* < 0.01) of the ipsilateral turns was developed by peripheral LPS pre-treated, compared with the PBS (i.p.) + PBS (striatum) group. Striatal vehicle injection [PBS (i.p.) group] made no differences in the behavior change at least 28 days compared with the control group.

These results clarified that endotoxic tolerance pre-treatment could improve intracranial LPS injection-induced behavioral impairment.

## Discussion

Neuroinflammation has been established as a crucial mechanism in neurodegeneration. Moreover, a growing body of evidence demonstrates that periphery immunity exerts significant impact on neuroinflammation. Our recent study has identified the key roles of PBM and microglia in inflammatory transmission from the periphery to the brain, demonstrating the strong influence of flamed peripheral inflammation on brain immune reactivity [14]. Immune tolerance restricts excessive inflammation and serves as a vital mechanism to avoid unnecessary by-stander injury, while monocyte/macrophage serves as the main cells responsible for the induction of immune tolerance in vivo [28]. Here, we investigated the effects of peripheral immune tolerance pre-treatment on neuroinflammation-induced neurodegeneration.

### Peripheral immune tolerance suppresses inflammatory activity of peripheral blood monocyte

Immune tolerance is a protective mechanism facing to excessive inflammation and unnecessary by-stander injury [29]. Endotoxic tolerance is always induced by a low dose of LPS and deemed as the most essential component of immune tolerance when suffering from subsequent inflammatory challenge [20, 30]. Our study indicated that repeated intraperitoneal injection of 0.3 mg/kg LPS for consecutive 4 days is indeed to induce the endotoxic tolerance of PBM characterized by decreased pro-inflammatory cytokines (TNF-α, IL-1β) and downregulated TLR4 expression under further LPS restimulation [31, 32]. Decreased inflammatory cytokines showed the peripheral immune tolerance induced-hypoactive state of PBM. TLR4 serve as the major pattern recognition receptors involved in the detection of LPS so that downregulation of TLR4 expression means diminished inflammatory response of PBM [33, 34]. This proves that PBM are the decisive cells in the formation of LPS-induced peripheral immune tolerance.

### Repeated low-dose LPS injection is failing to cause neuroinflammation or dopaminergic neuronal loss

Our previous study has proved that the peripheral high-dose LPS (5 mg/kg) injection could induce an inflammatory effect in the SN as demonstrated by the increase of pro-inflammatory cytokines and microglial activation [14], which was in line with the classical study carried out by Qin et al. [6]. However, the dose of LPS we used in the present study was considered to be a low-dose (0.3 mg/kg). Intracephalic immune status was monitored after repeated low-dose LPS injection-induced peripheral immune tolerance. The results indicated that repeated low-dose LPS intraperitoneal injection was insufficient to cause inflammation within the SN of healthy rats (there was neither an increase of inflammatory cytokines nor an upregulation of Iba-1). Previous studies demonstrate that the levels and distribution of inflammatory factors in the brain vary with the various dosage of peripheral LPS administration [4, 35]. Quan et al. confirmed that high dose of LPS induced global expression of pro-inflammatory cytokines in the brain, whereas low dose of LPS increased TNF-α and IL-1β mRNA expression only in the choroid plexus, circumventricular organs, and meninges without brain parenchyma [36]. This conclusion coincides with our results, although other regions outside the SN have not been measured in our study. In addition, we also observed the neuronal morphology and the total numbers of dopaminergic neurons in the SN after repeated intraperitoneal LPS administration. Our results revealed that the immunoreactivity of TH expressed on dopaminergic neurons was preserved in the SN.

### Peripheral immune tolerance pre-treatment alleviates the intracranial LPS injection-induced neuroinflammation

In the present study, we investigated the role of peripheral immune tolerance in the occurrence and progress of subsequent intracranial LPS injection-neuroinflammation. Our results showed that peripheral immune tolerance preconditioning attenuates the intracranial LPS injection-induced neuroinflammation, manifested as the decrease of inflammatory cytokines and the inhibition of microglial activation. This suggests that the regulation of PBM immune status could affect the progression of neuroinflammation [13]. We speculated that the neuroinflammatory alleviation was induced by peripheral immune tolerance preconditioning through the following ways: (a) Monocytes and microglia are considered to share the same origin of the myeloid lineage [37]; thus, immune tolerance pre-treatment induced hypo-responsiveness of monocytes may reflect the weakened immune activity of microglia in the brain correspondingly [38]. (b) Immune tolerant PBM may decrease the pro-inflammatory cytokines infiltrating from periphery into the brain correspondingly [39, 40]. (c) Peripheral immune tolerance may impair immune activity of PBM, which would migrate from the periphery into the brain through development into microglia directly or interacting with microglia indirectly to aggravate neuronal damage after neuroinflammation [41, 42]. (d) Peripheral immune tolerance may reshape the central microenvironment under neuroinflammation challenge by lessening interaction between PBM and other cells in the brain, such as perivascular cells [43] as well as endothelial cells of the BBB [44]. (e) Hypo-reactive PBM induced by peripheral immune tolerance may be associated with reduced T cellular infiltration into the brain [17, 45].

### Peripheral immune tolerance pre-treatment protects rats from neuroinflammation-induced neurodegeneration

The incidence of peripheral immune tolerance has been reported in several systemic disease settings, including sepsis, trauma, surgery, and pancreatitis, underlining its clinical significance [30]. In this study, we evaluated the effect of peripheral immune tolerance on neuroinflammation-related neurodegeneration, and our results suggested that LPS preconditioning could protect rats from neuroinflammation-induced neurodegeneration, showing as the increase of neuronal survival and the improvement of behavioral damage. Rosenzweig et al. clarified that LPS preconditioning modulates the cellular inflammatory response after cerebral ischemia, resulting in neuroprotection [21]. Besides, LPS preconditioning conferred neuroprotection has been proved in traumatic brain injury [22] and deep hypothermic circulatory arrest [23]. In accordance with these results, we observed that peripheral immune tolerant preconditioning-induced hypo-responsiveness of PBM led to a significant neuroprotection from subsequent neurodegeneration. It is sufficient to show that PBM serve as a significant regulator in the development and progression of neurodegenerative diseases.

It is recognized that excessive microglial activation can lead to the development of neurodegenerative diseases [9]; on the contrary, our present study showed that the neuroprotection of LPS preconditioning from neurodegeneration was accompanied by inhibition of microglial activation. Rodents that pre-treated with fluoxetine or somatostatin showed neuroprotective effects depending on inhibition of neuroinflammation-related microglial activation [46, 47]. These results highlight the neuroprotective role of microglial immunocompetent restriction in neuroinflammation-induced neurodegenerative diseases. Therefore, inhibition of microglial over-activation caused by peripheral immune tolerance produces neuroprotection.

Taken together, our study provides evidence that peripheral immune tolerance preconditioning play a crucial role in neuroprotection from subsequent neuroinflammation-induced neurodegeneration, and both PBM and microglia may serve as potential significant elements in this neuroprotection. Being different from previous studies, we regarded neurodegeneration as systemic diseases rather than disorders limited in the CNS. The results of our study may provide evidences for the immunologic communication between brain and periphery and likely produce an easier and more convenient peripheral way to manipulate the immunologic activities within the brain. However, our study is limited in that the study of molecular mechanisms and signaling pathways under this neuroprotection needs to be further implemented.

## Conclusions

Peripheral immune tolerance is an important regulatory mechanism for the immunological activity of PBM. Our results strongly indicated that peripheral immune tolerance attenuated subsequent neuroinflammation and provided neuroprotection from neuroinflammation-induced neurotoxicity, which will provide new therapeutic approaches for neurodegenerative diseases.

## Additional files

Additional file 1: Figure S1. Repeated intraperitoneal injection of 0.3 mg/kg LPS for 4 days induced peripheral immune tolerance of PBM. Peripheral blood monocytes of rats from the five groups pre-treated with PBS (0.3 ml/kg), LPS (0.1, 0.3, or 0.9 mg/kg) or without pre-treatment (control group) were isolated and cultured, and then, monocytes were restimulated by a single high-dose LPS (100 ng) for 4 h in vitro. The production of TNF-α and the expression of TLR4 in PBM were downregulated after LPS restimulation by repeated 0.3 mg/kg LPS intraperitoneal injections in 0.3 mg/kg LPS (i.p.) group, while the similar downregulation of inflammation was not observed in the other two groups with repeated 0.1 or 0.9 mg/kg LPS intraperitoneal injection. (a–b) The levels of TNF-α (a) and IL-1β (b) in the supernatant were detected by ELISA. (c–d) TLR4 production in PBM was quantified by measuring band intensities using ImageJ software. The values were normalized to GAPDH. **P < 0.01, ***P < 0.001 vs. control group. #*P* < 0.05, ##*P* < 0.01, ###*P* < 0.001 vs. PBS (i.p.) group. Data are presented as the mean ± SEM (*n* = 6). (TIFF 275 kb)

Additional file 2: Figure S2. Striatal injection of 15 μg LPS-induced generation and loss of dopaminergic neurons in the SN. A single dose of vehicle (5 μl PBS) or LPS (15 μg in 5 μl PBS) was administered to the right striatum in the PBS (striatum) group or in the LPS (striatum) group individually, and there was a control group without any treatment. The brains were removed at time point of 1, 7, 14, and 28 days after striatal injection, and the right SN was dissected out. The results implied that striatal injection of 15 μg LPS induced significant loss of dopaminergic neurons in the SN. (a) Immunohistochemistry staining on frozen sections of TH in the three groups of our observation. Since the control group and the PBS (striatum) group were not statistically significant at each time point, we selected control group as a representative. Moreover, the difference had not been distinguished as early as 7 days, so the data of the PBS (striatum) group and the LPS (striatum) group at 1 and 7 days was not shown. (b) Total numbers of TH-positive neurons in the injected side of SN (right SN) collected by stereological counting. ##*P* < 0.01 vs. PBS (striatum) group. Data are presented as the mean ± SEM (*n* = 3). Scale bars, 250 μm (40×), 100 μm (100×), and 25 μm (200×). (TIFF 3252 kb).

## Abbreviations

ANOVA: One-way analysis of variance
BBB: Blood-brain barrier
CNS: Central nervous system
ELISA: Enzyme-linked immunosorbent assay
ET: Endotoxic tolerance
i.p.: Intraperitoneal
IL-10: Interleukin-10
IL-1β: Interleukin-1β
LPS: Lipopolysaccharide
LSD: Least Significant Digit
PBM: Peripheral blood monocytes
PBS: Phosphate buffered saline
PD: Parkinson’s disease
PVDF: Polyvinylidene difluoride membranes
SEM: Standard error of the mean
SN: Substantia nigra
TGF-β: Transforming growth factor-β
TLR4: Toll-like receptor 4
TNF-α: Tumor necrosis factor-α
