# Supplementary figures and images for: Peripheral immune tolerance alleviates the intracranial lipopolysaccharide injection-induced neuroinflammation and protects the dopaminergic neurons from neuroinflammation-related neurotoxicity
Source: J Neuroinflammation. 2017 Nov 16;14:223. doi: 10.1186/s12974-017-0994-3 (PMC5693474; doi:10.1186/s12974-017-0994-3)

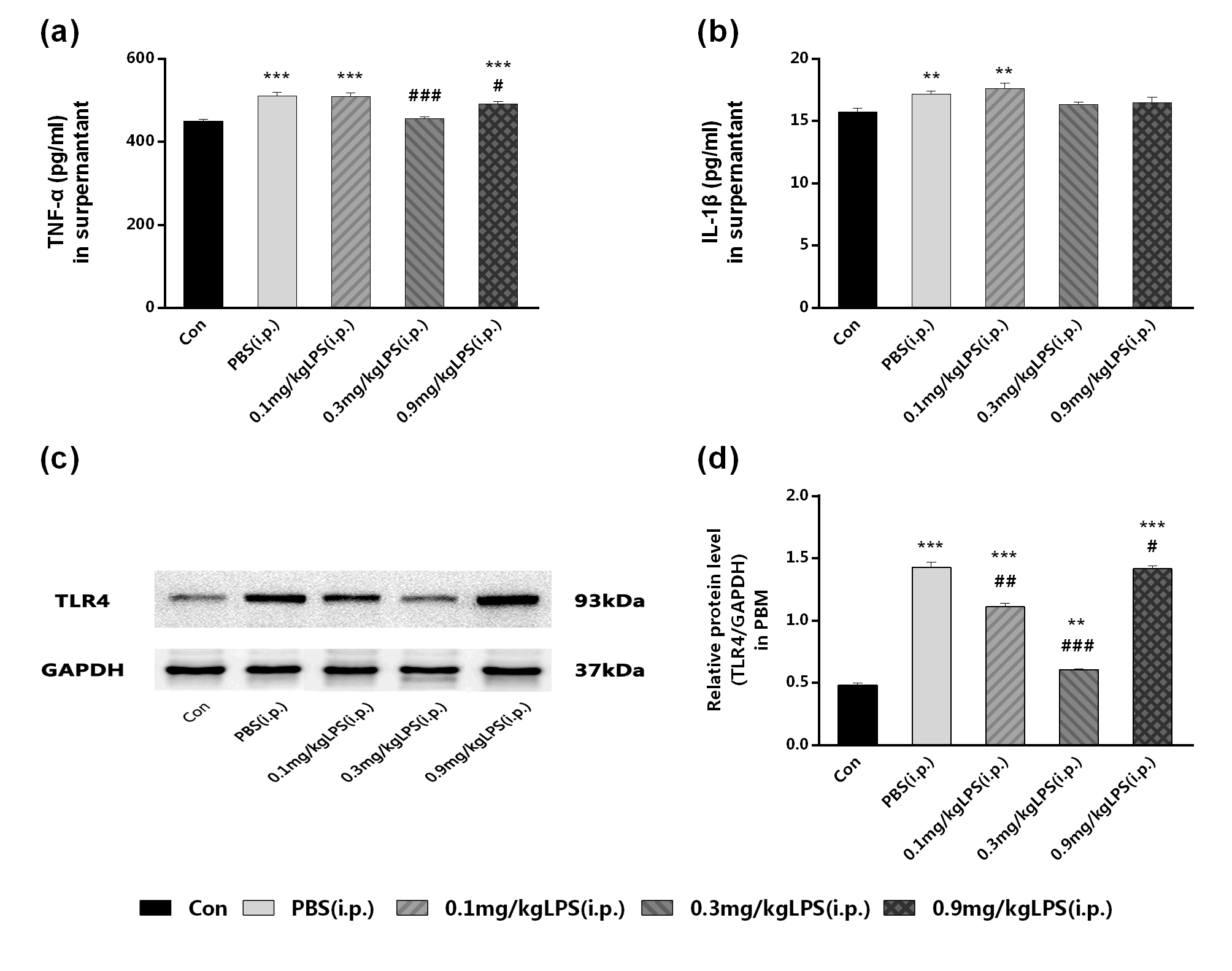

Supplement: Supplementary file 1 — Repeated intraperitoneal injection of 0.3 mg/kg LPS for 4 days induced peripheral immune tolerance of PBM. Peripheral blood monocytes of rats from the five groups pre-treated with PBS (0.3 ml/kg), LPS (0.1, 0.3, or 0.9 mg/kg) or without pre-treatment (control group) were isolated and cultured, and then, monocytes were restimulated by a single high-dose LPS (100 ng) for 4 h in vitro. The production of TNF-α and the expression of TLR4 in PBM were downregulated after LPS restimulation by repeated 0.3 mg/kg LPS intraperitoneal injections in 0.3 mg/kg LPS (i.p.) group, while the similar downregulation of inflammation was not observed in the other two groups with repeated 0.1 or 0.9 mg/kg LPS intraperitoneal injection. (a–b) The levels of TNF-α (a) and IL-1β (b) in the supernatant were detected by ELISA. (c–d) TLR4 production in PBM was quantified by measuring band intensities using ImageJ software. The values were normalized to GAPDH. **P < 0.01, ***P < 0.001 vs. control group. #P < 0.05, ##P < 0.01, ###P < 0.001 vs. PBS (i.p.) group. Data are presented as the mean ± SEM (n = 6). (TIFF 275 kb) [file 12974_2017_994_MOESM1_ESM.tif]

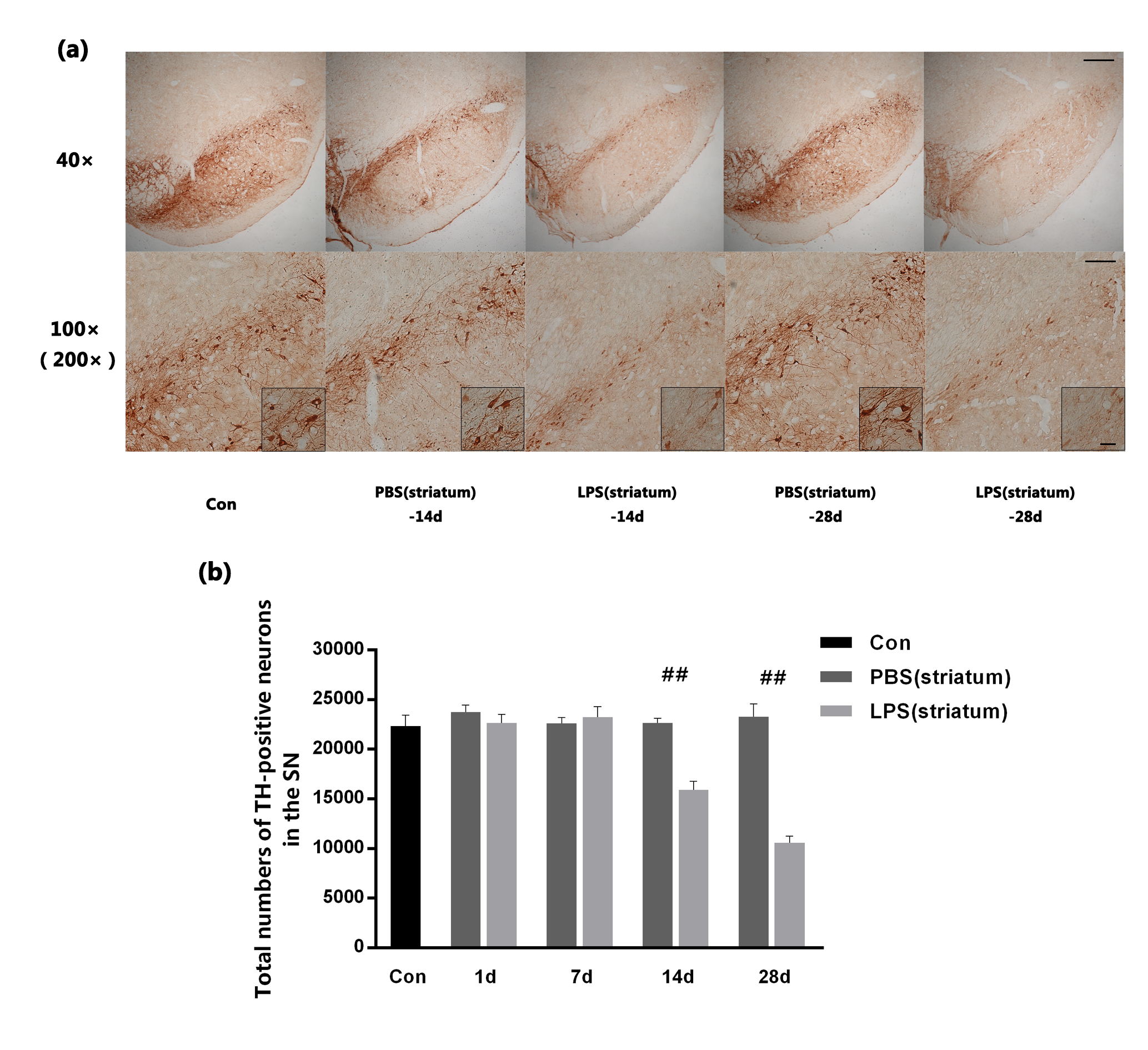

Supplement: Supplementary file 2 — Striatal injection of 15 μg LPS-induced generation and loss of dopaminergic neurons in the SN. A single dose of vehicle (5 μl PBS) or LPS (15 μg in 5 μl PBS) was administered to the right striatum in the PBS (striatum) group or in the LPS (striatum) group individually, and there was a control group without any treatment. The brains were removed at time point of 1, 7, 14, and 28 days after striatal injection, and the right SN was dissected out. The results implied that striatal injection of 15 μg LPS induced significant loss of dopaminergic neurons in the SN. (a) Immunohistochemistry staining on frozen sections of TH in the three groups of our observation. Since the control group and the PBS (striatum) group were not statistically significant at each time point, we selected control group as a representative. Moreover, the difference had not been distinguished as early as 7 days, so the data of the PBS (striatum) group and the LPS (striatum) group at 1 and 7 days was not shown. (b) Total numbers of TH-positive neurons in the injected side of SN (right SN) collected by stereological counting. ##P < 0.01 vs. PBS (striatum) group. Data are presented as the mean ± SEM (n = 3). Scale bars, 250 μm (40×), 100 μm (100×), and 25 μm (200×). (TIFF 3252 kb). [file 12974_2017_994_MOESM2_ESM.tif]
